# Zinc Supplementation Sustains Diaphragm Contractility and Preserves *SERCA2a* Expression in Aged Female Rats with Type 2 Diabetes

**DOI:** 10.3390/biom16091236

**Published:** 2026-08-26

**Authors:** Omer Unal, Nilufer Akgun-Unal

**Affiliations:** 1 Department of Physiology, Faculty of Medicine, Samsun University, Samsun 55080, Türkiye; 2 Department of Biophysics, Faculty of Medicine, Ondokuz Mayis University, Samsun 55139, Türkiye; nilufer.akgununal@omu.edu.tr

**Keywords:** diaphragm muscle, Type 2 diabetes, zinc sulfate, isometric contraction, *SERCA2a*

## Abstract

Diabetes mellitus (DM) is a chronic metabolic disease characterized by hyperglycemia, and the diaphragm—the primary respiratory muscle—is adversely affected by this diabetic process. The aim of this study is to investigate the effects of zinc sulfate (ZnSO_4_) treatment on diaphragm muscle contractile dynamics, calcium homeostasis, apoptosis, and fibrosis in an 18-month-old female Type 2 diabetic rat model. Thirty-two 18-month-old female Wistar rats were randomly divided into four groups: Control (CON), CON + ZnSO_4_, Diabetes Mellitus (DM), and DM + ZnSO_4_. The DM model was induced by a high-fat diet and administration of 30 mg/kg streptozotocin (STZ); after the disease was confirmed, ZnSO_4_ was administered intraperitoneally at a daily dose of 10 mg/kg to the treatment groups. The mechanical functions of the diaphragm muscle were evaluated using a post-rest potentiation protocol in an isolated organ bath; qPCR analyses (*Caspase-3*, *TGF-β1*, *SERCA2a*) were performed to investigate cellular apoptosis, fibrosis, and calcium regulation. Compared with the CON group, the DM group exhibited a severe ~90% reduction in diaphragmatic contraction force (CF) and a ~97% decline in maximal contraction/relaxation velocities (±dF/dt_max_) (*p* < 0.0001), which strongly correlated with a 30% suppression of *SERCA2a* gene expression (*p* < 0.01). Concomitantly, apoptotic *Caspase-3* (~2.6-fold) and profibrotic *TGF-β1* (~3.1-fold) mRNA levels were significantly elevated (*p* < 0.0001). In the DM + ZnSO_4_ group, daily zinc treatment (10 mg/kg/day, i.p. for 6 weeks, initiated 4 weeks after diabetes confirmation) did not reverse the elevated *Caspase-3* and *TGF-β1* expressions (*p* > 0.05). However, *SERCA2a* expression was fully preserved back to control levels (*p* < 0.05 vs. DM), leading to a substantial ~3-fold improvement in CF and velocities (*p* < 0.05 to *p* < 0.0001 vs. DM). On the other hand, the healthy CON + ZnSO_4_ group exhibited a physiological slowing of contractility (~53% decrease in CF), without histological damage, likely due to a competitive antagonism between excess divalent zinc (Zn^2+^) and calcium (Ca^2+^) on myofilaments. Although zinc cannot reverse the structural apoptotic and fibrotic remodeling in the aged diabetic diaphragm, it successfully rescues functional contractility by preserving *SERCA2a* transcriptional expression.

## 1. Introduction

DM is a chronic metabolic disease characterized by hyperglycemia [1,2]. Although the cardiovascular and renal complications of diabetes have been extensively studied in the literature [3,4], its effects on skeletal muscles have received relatively less attention. The diaphragm, the primary respiratory muscle in mammals, is one of the major skeletal muscles affected by the diabetic process [5]. In the diaphragm, which is engaged in continuous contraction throughout life, it has been reported that oxidative stress caused by diabetes-related hyperglycemia and increased reactive oxygen species (ROS) disrupts muscle function and leads to changes in action potentials [6]. Increased oxidative stress, by disrupting the physiological balance of the cell membrane and calcium (Ca^2+^) homeostasis, can lead to dysfunction in the diaphragm’s isometric contraction parameters [7]. Furthermore, it has been shown that fibrosis—characterized by collagen accumulation and inflammation in diabetic diaphragmatic tissue—adversely affects this cellular function [5].

Increased oxidative stress and subsequent apoptosis accelerate diaphragmatic tissue damage by disrupting intracellular homeostasis [8]. In addition to apoptosis, the release of pro-inflammatory cytokines paves the way for tissue fibrosis. In this fibrotic process, the activation of the Transforming Growth Factor-Beta 1 (TGF-β1) pathway plays a significant role [9].

Zinc (Zn), one of the essential trace elements in the body, plays a regulatory role in physiological processes such as redox balance, immune response, and cell survival [10]. Zinc deficiency is commonly observed in individuals with diabetes due to increased urinary zinc excretion [11]. In vivo studies in the literature have shown that exogenous zinc supplementation has protective effects against diabetic cardiomyopathy, nephropathy, and liver damage [8,12]. These effects of zinc are explained by its activation of antioxidant pathways (e.g., Nrf2), inhibition of caspase activation to halt apoptosis, and suppression of *TGF-β1* production to limit fibrosis [8,9].

However, the effects of zinc on skeletal muscle, particularly when combined with the factor of advanced age, are not yet fully understood. The aging process is characterized by a loss of function and mass in skeletal muscles. The presence of diabetes in older age increases oxidative stress and cellular degradation processes in continuously working muscles such as the diaphragm. A recent study reported that zinc sulfate (ZnSO_4_) administered to 18-month-old female rats with Type 2 diabetes provided protection by suppressing oxidative damage in cardiac tissue, although it may cause mild stress in healthy myocardium [13]. Although it is known that zinc protects tissues in aged diabetic models, its anti-apoptotic and anti-fibrotic effects in diaphragm tissue within a model combining aging and diabetes have not been clearly elucidated. The selection of 18-month-old female rats was scientifically reasoned to address specific clinical and ethical gaps. Clinically, Type 2 diabetes mellitus and diaphragmatic weakness are progressive conditions that predominantly affect elderly populations, where they interact synergistically with age-related muscle sarcopenia. While most preclinical studies utilize young adult rodents (typically 8–12 weeks of age), they fail to replicate the complex pathophysiological environment of the aged diaphragm. Additionally, 18-month-old female rats exhibit estropause (ovarian senescence), which provides a clinically relevant model for postmenopausal estrogen depletion—a state known to independently exacerbate skeletal muscle wasting, oxidative stress, and calcium dysregulation.

Accordingly, the aim of this study is to investigate the potential protective effects of intraperitoneal (i.p.) ZnSO_4_ treatment on the diaphragm muscle in an 18-month-old female rat model of Type 2 diabetes. The study evaluated the histopathological changes in the diaphragm caused by diabetes, cellular apoptosis levels, and the development of fibrosis; it also assessed whether zinc treatment could correct the functional impairments in isometric contraction and sarcoplasmic reticulum (SR) calcium dynamics that accompany these structural damages. We hypothesized that daily zinc supplementation would rescue diabetic-induced diaphragmatic contractile dysfunction by preserving the transcriptional expression of the sarcoplasmic reticulum Ca^2+^-ATPase (*SERCA2a*) pump, even in the presence of ongoing structural apoptotic and profibrotic pathways.

## 2. Materials and Methods

### 2.1. Experimental Animals and Study Groups

This study complied with national and international guidelines on animal welfare and utilized the ARRIVE guidelines to ensure transparent and reliable reporting of research involving live animals. All experimental protocols and procedures were approved by the Ethics Committee of the Faculty of Medicine at KTO Karatay University (Approval No.: 2026/032, 21 May 2026). A total of 32 18-month-old female rats were used in the study. The characteristics of this animal cohort, including type 2 diabetes induction and zinc sulfate administration protocols, were performed as previously described [13]. The animals were housed in rooms maintained at standard laboratory temperature (21 ± 1 °C) with a 12-h light/dark cycle and had free access to food and water. All rats were randomly divided into four equal groups (*n* = 8), which were defined as follows: Control (CON): Healthy aged rats fed a standard diet. On the day of diabetes induction, a single dose of 0.1 M citrate buffer (pH 4.4) was administered instead of STZ, and normal saline was administered intraperitoneally (i.p.) daily throughout the treatment period. Control + Zinc Sulfate (CON + ZnSO_4_): This group was fed a standard diet and received a citrate buffer instead of STZ. Throughout the treatment phase, the animals were administered zinc sulfate (ZnSO_4_) at a daily dose of 10 mg/kg i.p [13]. Diabetes Mellitus (DM): This group was fed a high-fat diet (HFD) to establish a Type 2 diabetes model and received a single 30 mg/kg i.p. dose of STZ after a 12-h fast. Animals with fasting blood glucose levels >250 mg/dL were considered diabetic and received daily i.p. saline during the treatment phase [14,15]. Diabetes + Zinc Sulfate (DM + ZnSO_4_): This group was induced to become diabetic via the HFD and 30 mg/kg STZ procedure. After the disease model was confirmed, the animals were administered ZnSO_4_ at a daily dose of 10 mg/kg i.p. throughout the treatment period.

### 2.2. Experimental Design and Timeline

**Scheme 1:**
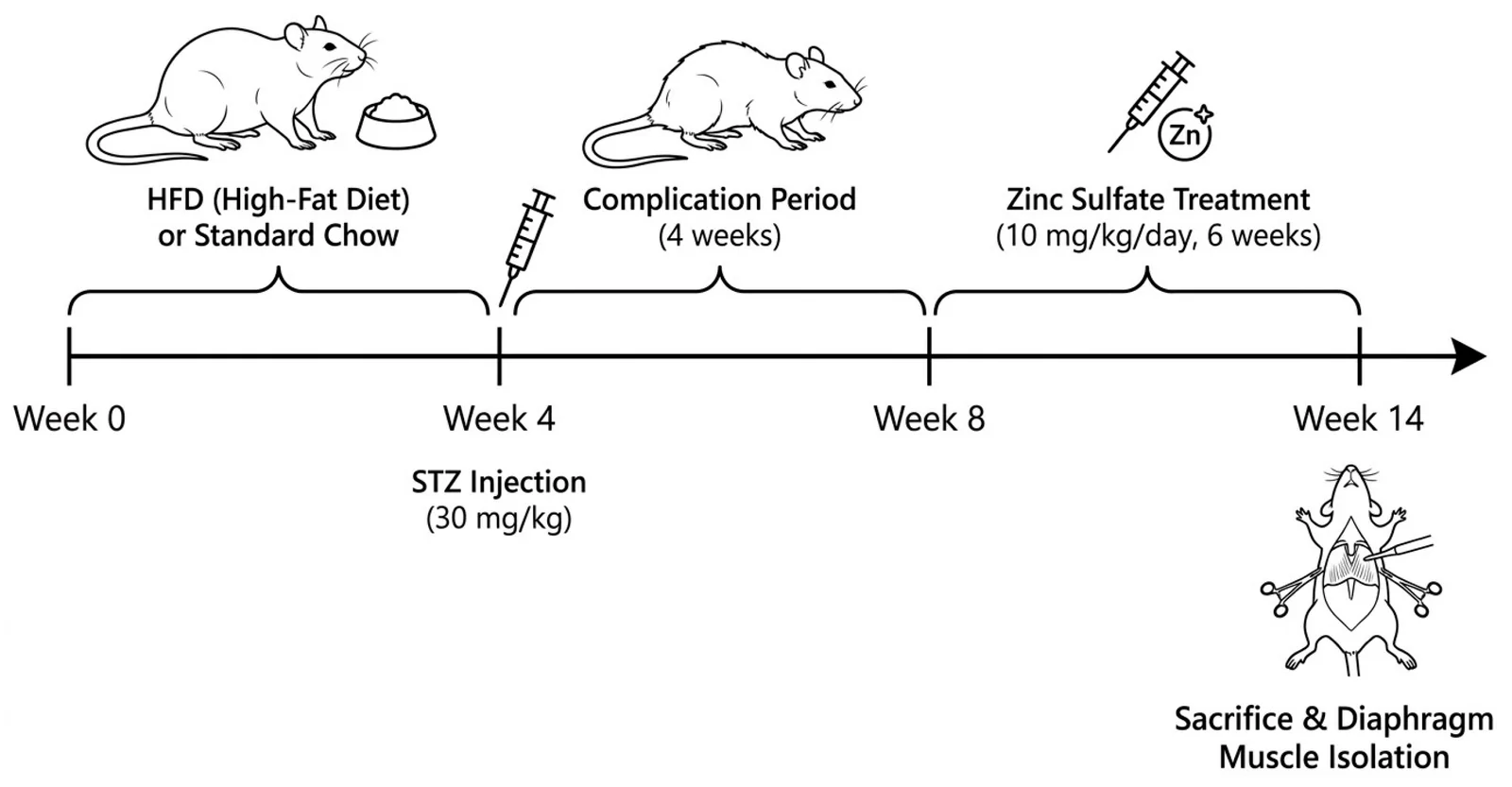
Schematic diagram of the experimental timeline and treatment protocol in aged female rats. The experimental design represents the timeline of high-fat diet (HFD) feeding, induction of Type 2 Diabetes Mellitus (T2DM) with a single dose of streptozotocin (STZ, 30 mg/kg, i.p.) at week 4, and the subsequent 6-week intraperitoneal (i.p.) treatment phase with zinc sulfate (ZnSO_4_, 10 mg/kg/day) or saline until sacrifice.

The overall experimental timeline spanned a total of 14 weeks (Scheme 1). Aged female Wistar rats in the DM and DM + ZnSO_4_ groups were first placed on a high-fat diet (HFD; 40% of calories from fat) for 4 weeks to induce obesity and peripheral insulin resistance, while CON and CON + ZnSO_4_ groups received standard rodent chow. At Week 4, following a 12-h fast, DM and DM + ZnSO_4_ groups received a single intraperitoneal (i.p.) injection of streptozotocin (STZ; 30 mg/kg dissolved in 0.1 M cold citrate buffer, pH 4.4). Nondiabetic control groups received an equivalent volume of citrate buffer. Fasting blood glucose (FBG) levels were evaluated 72 h post-STZ administration via tail vein blood samples using a validated glucometer. Rats displaying FBG levels > 250 mg/dL were confirmed as successfully induced diabetic animals. The combination of a high-fat diet (HFD) and a low-dose STZ (30 mg/kg) injection is a well-established and widely accepted experimental paradigm that mimics the pathogenesis of human Type 2 diabetes mellitus (T2DM). While a single high dose of STZ (>50 mg/kg) on standard chow is classically used to induce Type 1 diabetes via absolute beta-cell destruction, the pre-treatment with HFD induces peripheral insulin resistance and dyslipidemia, and the subsequent sub-diabetogenic low-dose STZ selectively impairs pancreatic insulin secretion without causing total insulinopenia, thereby replicating the clinical phenotype of late-stage T2DM.

Following diabetic confirmation, all rats underwent a 4-week complication period (Weeks 4 to 8) without pharmacological intervention, allowing the full development of chronic diabetic cardiovascular and skeletal muscle abnormalities. At Week 8 (exactly 4 weeks after diabetes confirmation), the treatment phase was initiated and continued daily for 6 weeks (Weeks 8 to 14). During this phase, rats in the CON + ZnSO_4_ and DM + ZnSO_4_ groups received daily i.p. injections of zinc sulfate (ZnSO_4_, 10 mg/kg/day), while CON and DM groups received an equivalent volume of physiological saline. At Week 14, body weights and final FBG levels were measured, and all animals were sacrificed concurrently for fresh diaphragm tissue isolation.

### 2.3. Histological Processing

Diaphragm tissues dissected from rats were fixed in a 2% paraformaldehyde + 2% glutaraldehyde solution and then subjected to routine histological processing procedures. They were then embedded in L-shaped plates and labeled blindly. Sections 5 µm thick, obtained from the tissues using a microtome, were used for histopathological evaluation, and sections 4 µm thick were used for immunohistochemical evaluation. For histopathological evaluation, sections were stained with hematoxylin and eosin. Immunohistochemical staining was performed using the HRP/AEC Detection IHC Kit (ab93705, Abcam, Cambridge, MA, USA). Images were captured using a light microscope equipped with a camera attachment (Olympus, BX43, Center Valley, PA, USA). Tissues were incubated overnight at +40 °C with the primary antibody anti-active-caspase-3 (dilution: 1/200, orb500767, Biorbyt Ltd., San Francisco, CA, USA). To ensure objectivity and reproducibility, the semi-quantitative scoring of histopathological and immunohistochemical changes was performed by three independent histologists blinded to the experimental groups. Fibrosis was evaluated in H&E-stained sections based on the following structural criteria: Score 0 (no fibrosis) was assigned to normal connective tissue distribution without expansion of the perimysium or endomysium; Score 1 (slight) represented focal, localized expansion of the perimysium with minimal collagen deposition; Score 2 (moderate) indicated diffuse expansion of both endomysial and perimysial spaces with clear separation of myofibers; and Score 3 (severe) represented extensive connective tissue deposition replacing degenerated muscle fibers. Apoptosis was assessed via active *Caspase-3* immunoreactivity based on the percentage of positive-stained myofibers: Score 0 (negative) for <5% positive fibers; Score 1 (slight) for 5–25% positive fibers; Score 2 (moderate) for 26–50% positive fibers; and Score 3 (severe) for >50% positive fibers displaying intense immunoreactivity [16].

### 2.4. Molecular Analyses (qPCR)

Immediately following the sacrifice of the experimental animals, diaphragm muscle tissues were rapidly dissected and preserved. Total RNA extraction from frozen diaphragm samples was performed using a commercial isolation kit (Cat. No.: BS88203, Bio Basic, Markham, ON, Canada) in accordance with the manufacturer’s protocols. The concentration and purity of the obtained RNA were assessed spectrophotometrically using a microplate reader (Multiskan SkyHigh, Thermo Fisher, Waltham, MA, USA). Subsequently, the purified RNA samples isol were converted into complementary DNA (cDNA) using a high-throughput reverse transcription kit (Cat. No.: 4368813, Thermo Fisher, Vilnius, Lithuania) in a thermal cycler (ProFlex PCR System, Thermo Fisher, Waltham, MA, USA).

qPCR analysis was performed to quantitatively determine the mRNA expression levels of the target genes (*Caspase-3*, *TGF-β1*, and *SERCA2a*) in this study. Amplification reactions were set up in a total volume of 20 µL using a fluorescent master mix (FastStart Essential DNA Green Master, Cat. No.: 06 402 712 001, Roche, Basel, Switzerland), with three replicates (triplicates) for each sample. The specific primer sequences used for amplifying the target genes were commercially obtained (Oligomer, Ankara, Turkey) (Table 1). *GAPDH* was selected as the internal reference (housekeeping) gene for standardizing the qPCR data. The relative fold changes in gene expression were calculated using the standard 2^−ΔΔ*Ct*^ formula [17].

### 2.5. Isolated Organ Bath and Isometric Contraction Records

An isolated organ bath system was used to evaluate the mechanical contraction dynamics of the diaphragm muscle and the functions of the sarcoplasmic reticulum (SR). Immediately after the rats were sacrificed, muscle strips approximately 2–3 mm wide were harvested from the costal region of the diaphragm and suspended in organ baths containing Krebs–Henseleit solution (pH 7.4), maintained at 37 °C and continuously aerated with 95% O_2_/5% CO_2_ (carbogen). While one end of the muscle strips was fixed, the other end was attached to an isometric force transducer. An adaptation period of 60 min was provided by applying optimal resting tension to the tissues. Following adaptation, baseline isometric contraction recordings were obtained at a frequency of 1 Hz; additionally, a post-rest potentiation protocol was applied to the tissues to assess intracellular SR calcium (Ca^2+^) storage and release capacity. Under this protocol, electrical stimulation applied to muscle strips was interrupted for durations of 10, 20, 30, 40, 50, and 60 s, respectively, and the isometric responses generated by the muscle were recorded immediately upon the end of each rest period. To ensure technical standardization and control for biological variation in muscle size, all diaphragmatic strips were dissected to a uniform width of approximately 2.0 mm, parallel to the longitudinal axis of the muscle fibers, extending from the central tendon to the costal rib margin. After mounting the strips vertically in the organ bath, a resting tension (preload) of 1.0 g was systematically applied using a micro-positioner to stretch the muscles to their optimal length (L0) for force generation, followed by a 45-min equilibration period. At the completion of all stimulation protocols, the active muscle segment between the upper and lower suture ties was carefully excised, gently blotted on high-grade filter paper to remove excess Krebs–Henseleit buffer, and immediately weighed on a high-precision analytical balance (accuracy of 0.1 mg). The absolute isometric force (measured in grams, g) was then divided by the wet weight of the active muscle strip (measured in milligrams, mg) to normalize the contractility data, expressed as g/mg (or g·mg^−1^). The obtained isometric contraction force (g·mg^−1^), maximum contraction velocity (+dF/dtmax, g·mg^−1^·s^−1^), maximum relaxation velocity (−dF/dtmax, g·mg^−1^·s^−1^), and contraction power (AUC g·mg^−1^·s) were analyzed using a computer-based data acquisition system [18].

### 2.6. Statistical Analysis

All quantitative data are presented as mean ± standard deviation (SD). Statistical analyses were performed using GraphPad Prism software (Version 9.0, GraphPad Software Inc., San Diego, CA, USA). Since the experimental design followed a 2 × 2 factorial layout, data were analyzed using Two-way Analysis of Variance (ANOVA) to evaluate the main effects of the diabetic state (control vs. diabetic), treatment state (untreated vs. zinc-treated), and their interaction. Post-hoc pairwise comparisons were conducted using Tukey’s multiple comparison test. Histopathological and immunohistochemical semi-quantitative scores were analyzed using the non-parametric Kruskal–Wallis test followed by Dunn’s post-hoc test. A *p*-value of less than 0.05 was considered statistically significant.

## 3. Results

### 3.1. Metabolic Phenotypes and Animal Characteristics

To verify the successful induction of type 2 diabetes and evaluate the systemic influence of zinc sulfate, metabolic parameters including body weight and fasting blood glucose (FBG) were evaluated (Table 2).

Following STZ injection, DM and DM + ZnSO_4_ rats exhibited severe and stable hyperglycemia (>340 mg/dL) compared with euglycemic control groups (*p* < 0.001). At the end of the 14-week protocol, untreated diabetic rats (DM) displayed significant cachectic body weight loss and elevated FBG (395.4 ± 22.8 mg/dL). Daily treatment with ZnSO_4_ for 6 weeks significantly limited diabetes-induced body weight wasting (*p* < 0.01 vs. DM) and partially but significantly ameliorated hyperglycemia in DM + ZnSO_4_ rats (262.1 ± 19.3 mg/dL, *p* < 0.01 vs. DM group), confirming the active therapeutic and insulinomimetic role of zinc supplementation.

### 3.2. Histopathological Findings

**Figure 1:**
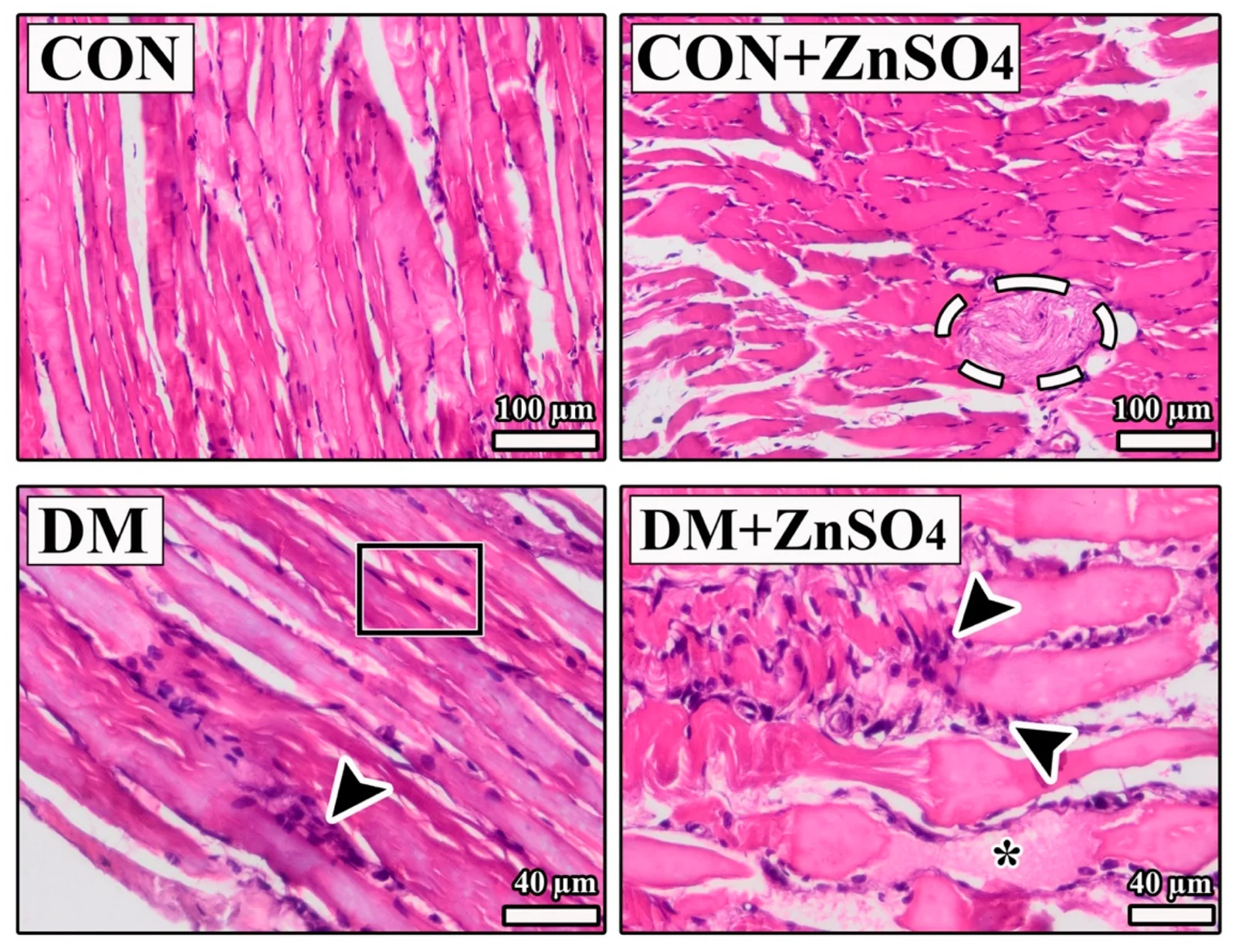
Images obtained from hematoxylin-eosin-stained sections of diaphragm tissue from the experimental groups. In the CON group, muscle fibers are normally structured and arranged in parallel. Muscle fiber nuclei have normal morphology. In the CON + ZnSO_4_ group, muscle fibers are seen to be normally structured and arranged, and a cross-section of the nerve bundle in the transverse plane is observed within the dashed circle. Although slight separations between fibers are observed in this group, no significant damage is evident. In the DM group, inflammation (arrowhead) and fiber separations are observed (area within the square). Increased interstitial connective tissue is observed in some areas of this group. In the DM + ZnSO_4_ group, areas of infiltration (arrowheads), localized disruption of the fiber structure, and interstitial expansion (*) are observed. Scale bars: 100 µm and 40 µm.

**Figure 2:**
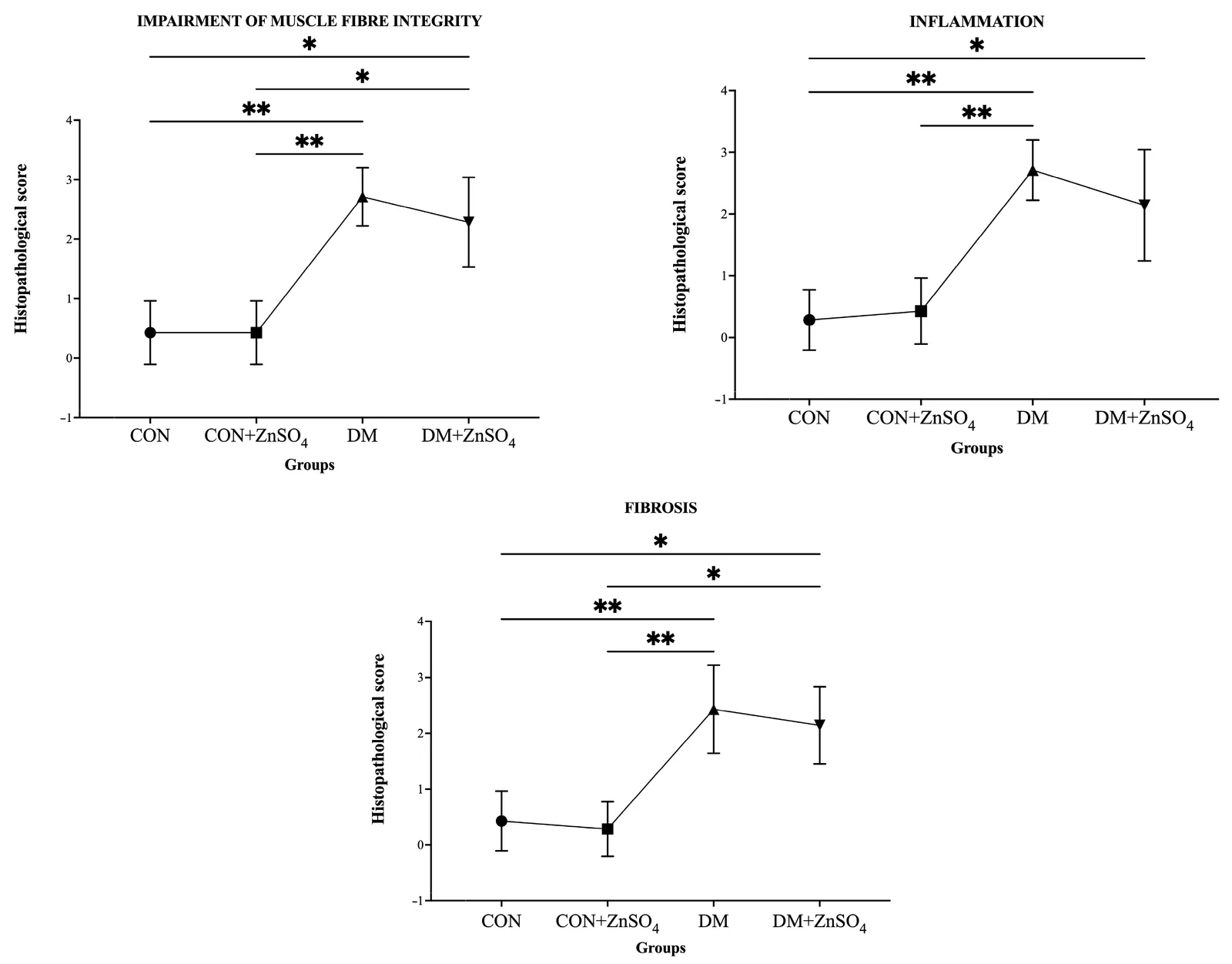
Semi-quantitative histopathological scoring of diaphragms across all groups for Impairment of muscle fiber integrity, Inflammation, and Fibrosis. Data are presented as mean ± SD. Statistical comparisons between groups were analyzed using the non-parametric Kruskal–Wallis test followed by Dunn’s post-hoc multiple comparison test. Significant differences are indicated by the comparison brackets: ** *p* < 0.01 indicates highly significant differences when comparing DM vs. CON and DM vs. CON + ZnSO_4_; * *p* < 0.05 indicates significant differences when comparing DM + ZnSO_4_ vs. CON and DM + ZnSO_4_ vs. CON + ZnSO_4_. Comparisons between CON vs. CON + ZnSO_4_, and DM vs. DM + ZnSO_4_ showed no statistically significant differences (*p* > 0.05).

Upon examining the images of the diaphragm muscle in all groups, it is observed that in the CON group, the fiber structure is healthy, and the interstitial spaces between the fibers are not open. On the other hand, the core structures are preserved. However, in the CON + ZnSO4 group, sections show gaps between fibers in some areas, but the fiber organization remains regular, and structural integrity is preserved. In the DM group, significant differences in fiber structure, vacuolated areas, and foci of infiltration are observed in some areas. In contrast, in the DM + ZnSO_4_ group, expanded interstitial connective tissue areas, regions of dense infiltration, and irregular fiber structures are also observed (Figure 1 and Figure 2).

### 3.3. Immunohistochemistry Findings

**Figure 3:**
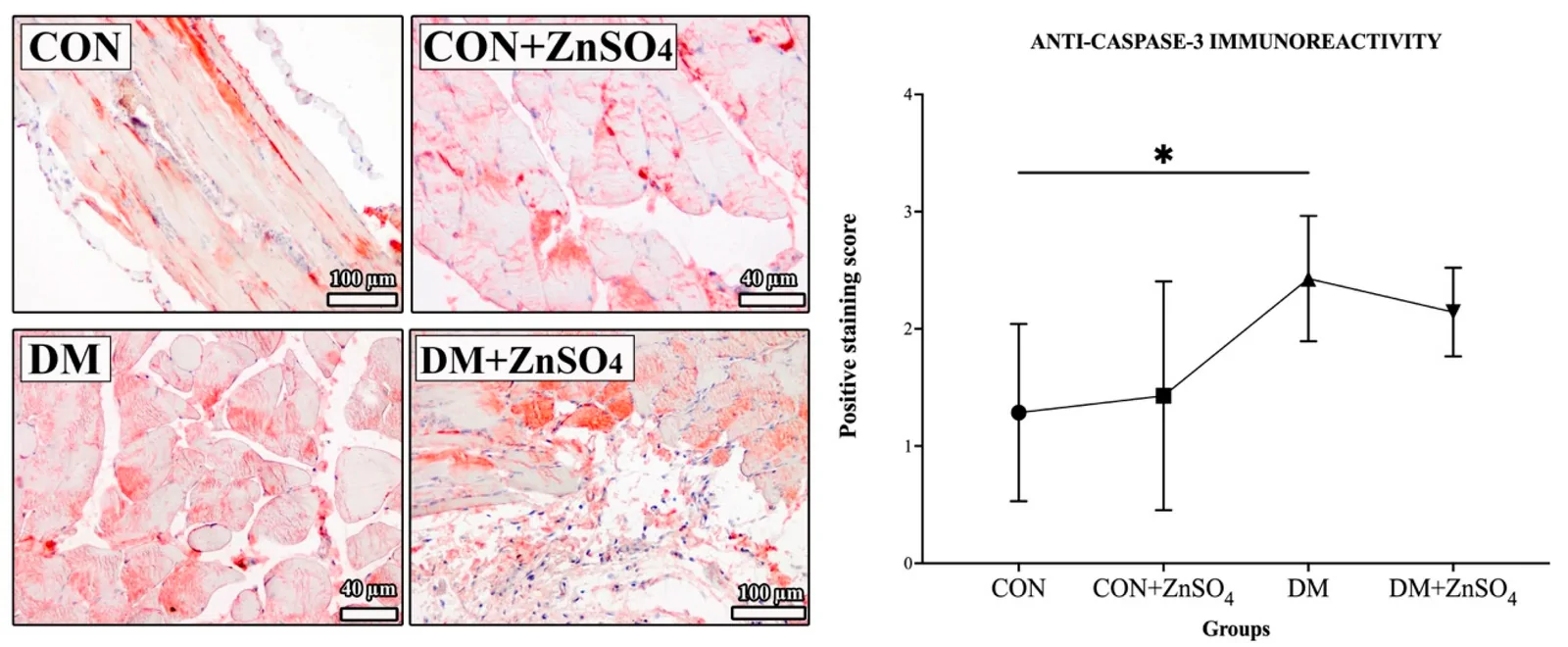
Anti-caspase-3 immunoreactivity was examined to assess apoptotic activity in sections from all groups. Significantly positive staining was observed in muscle fibers in the DM group. On the other hand, severe staining was observed in the DM + ZnSO_4_ group. However, the immunohistochemical score was significantly higher in the DM group than in the CON group. Scale bars: 100 µm and 40 µm. Mayer’s hematoxylin was used for counterstaining. The area stained brown-brick red indicates a positive reaction. Data were analyzed using Kruskal–Wallis followed by Dunn’s post-hoc test. *: *p* < 0.05.

**Figure 4:**
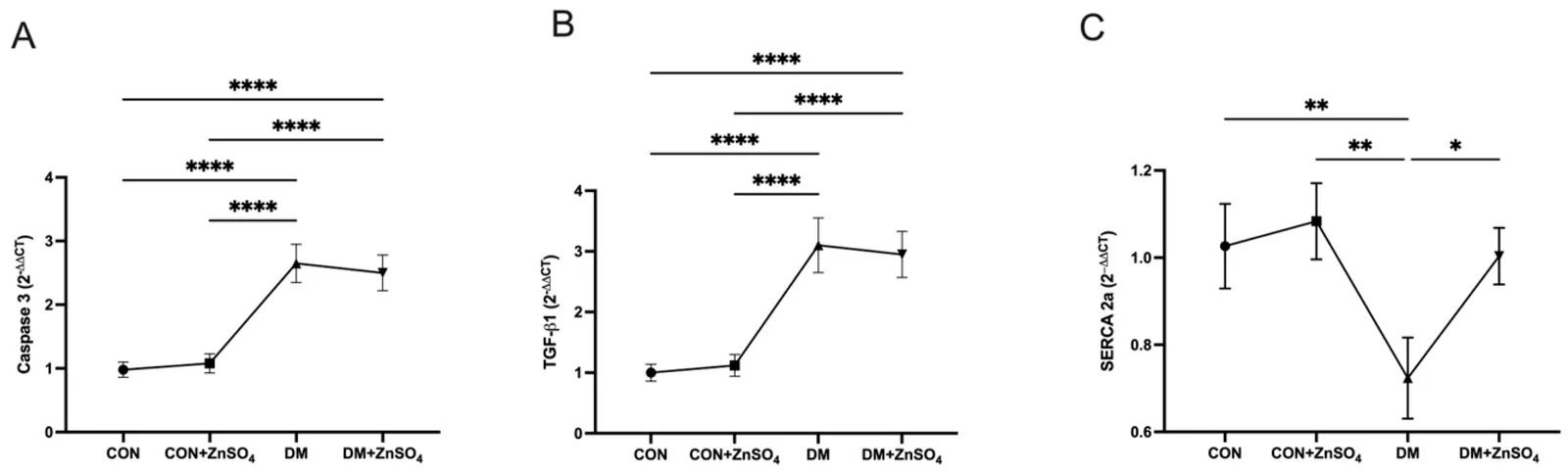
mRNA expression levels of *Caspase-3* (**A**), *TGF-β1* (**B**), and *SERCA2a* (**C**) in diaphragm muscle tissue across experimental groups. Relative fold changes in gene expression were calculated using the 2^−ΔΔ*Ct*^ formula, and data are presented as mean ± SEM. In the DM and DM + ZnSO_4_ groups, *Caspase-3* and *TGF-β1* gene expression showed a significant increase (**** *p* < 0.0001) compared with the CON and CON + ZnSO_4_ groups, and zinc treatment did not alter this increase. In contrast, *SERCA2a* expression, which was significantly suppressed in the DM group (** *p* < 0.01), was statistically significantly preserved in the DM + ZnSO_4_ group following zinc treatment (* *p* < 0.05). Data were analyzed using two-way ANOVA followed by Tukey’s post-hoc test.

Anti-caspase-3 immunoreactivity was evaluated in images obtained from all groups. A significant increase in anti-caspase-3 activity was observed in the DM group compared with the CON group (*p* < 0.05) (Figure 3). In the DM + ZnSO4 group, intense positive staining persisted within the muscle fibers. Although the semi-quantitative scoring did not reveal a statistically significant difference between the DM + ZnSO_4_ group and the CON group (*p* > 0.05) due to the inherent statistical limitations of the non-parametric post-hoc test, the apoptotic score remained notably high and did not show any significant reduction compared with the untreated DM group (*p* > 0.05). This persistent apoptotic activation in both diabetic cohorts is fully corroborated by our quantitative qPCR analysis (Figure 4A), which demonstrates highly significant elevations in *Caspase-3* mRNA expression compared with the CON group (*p* < 0.0001).

### 3.4. qPCR Findings

To evaluate the molecular mechanisms underlying diabetes-induced apoptosis, fibrosis, and Ca^2+^ homeostasis in diaphragm tissue, mRNA expression levels of the *Caspase-3*, *TGF-β1*, and *SERCA2a* genes were analyzed by qPCR, and the results are presented in Figure 4.

When the gene expressions of *Caspase-3*—the primary executor of cellular apoptosis—and TGF-β1—the master upstream signaling cytokine orchestrating the profibrotic cascade—were examined, no significant difference was detected between the CON and CON + ZnSO_4_ groups (*p* > 0.05). In contrast, in the DM group, the expression of both genes was statistically significantly elevated compared with the CON group (*p* < 0.0001). In the DM + ZnSO_4_ group, where zinc treatment was administered to the diabetic model, no significant decrease in *Caspase-3* and *TGF-β1* mRNA levels was observed compared with the DM group (*p* > 0.05). This finding corroborates our histopathological findings, indicating that zinc is insufficient in suppressing diabetes-associated apoptotic and upstream profibrotic gene activation.

On the other hand, a different picture emerged when evaluating the expression of *SERCA2a*, which plays a critical role in contraction dynamics. In the DM group, *SERCA2a* gene expression was found to be significantly suppressed and statistically significantly reduced compared with the CON group (*p* < 0.01). However, in the treated DM + ZnSO_4_ group, it was determined that the reduced *SERCA2a* expression was significantly preserved, and cellular levels had rebounded compared with the DM group (*p* < 0.05). These genetic findings provide molecular evidence that, even if ZnSO_4_ treatment cannot reverse structural damage (apoptosis and fibrosis) in the tissue, it supports the calcium cycle by preserving *SERCA2a* expression and improves mechanical contraction dysfunction in the diaphragm at the functional level.

### 3.5. Diaphragm Muscle Isometric Contraction Findings

**Figure 5:**
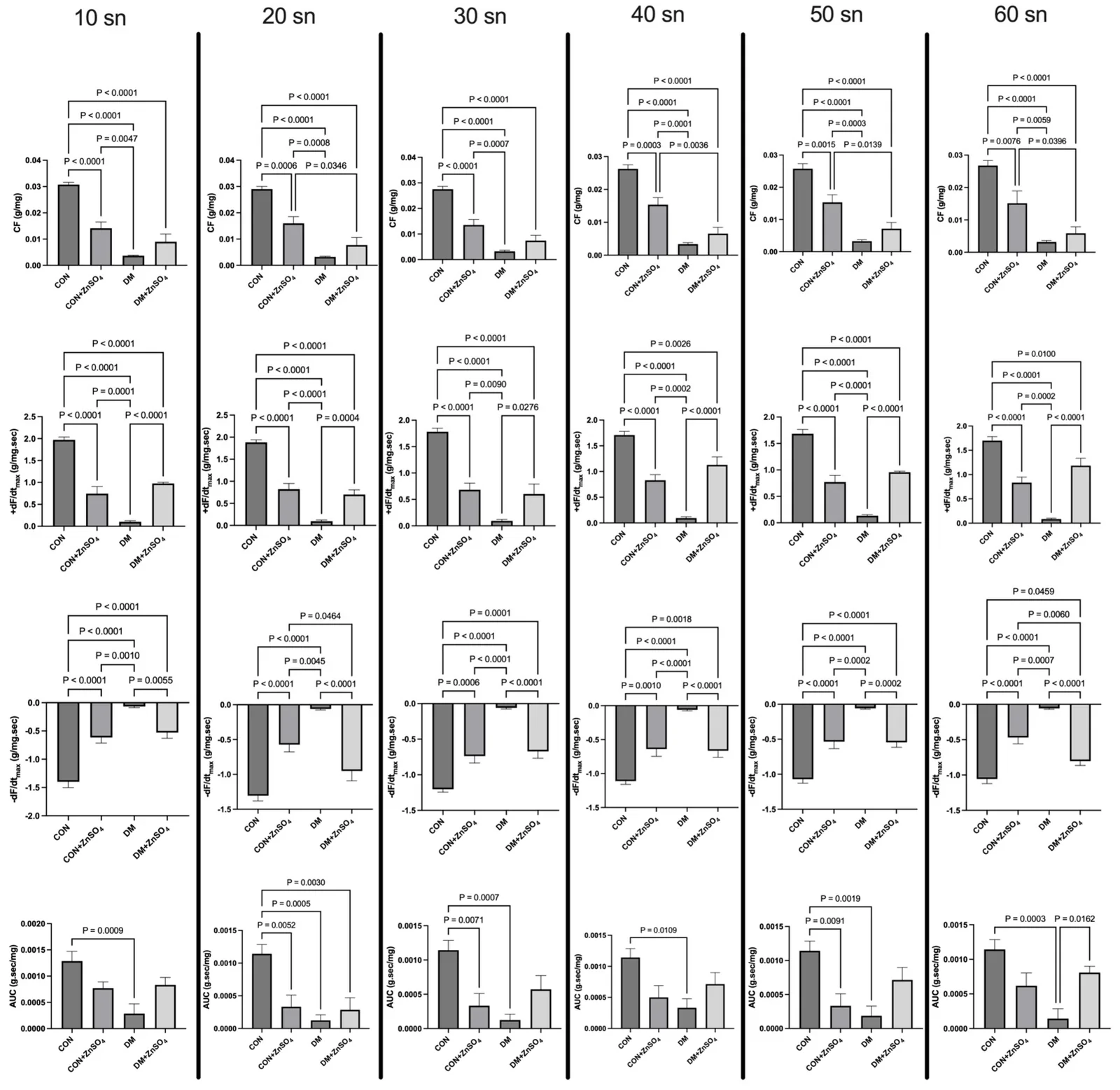
Isometric contraction parameters of the post-rest potentiation protocol applied to diaphragm muscle preparations at 10, 20, 30, 40, 50, and 60 s of resting times. Parameters include: contraction force (CF), maximum contraction velocity (+dF/dtmax), maximum relaxation velocity (−dF/dtmax), and contraction power (AUC). Abbreviations: Control (CON), Control + Zinc Sulfate (CON + ZnSO_4_), Diabetes Mellitus (DM), and Diabetes Mellitus + Zinc Sulfate (DM + ZnSO_4_). Intergroup comparisons were analyzed using two-way ANOVA followed by Tukey’s post-hoc test. Data are presented as mean ± SEM. Statistical significance for all pairwise comparisons is systematically indicated as follows: (1) DM vs. CON: A highly significant decrease was observed in all parameters (CF, +dF/dtmax, −dF/dtmax, and AUC) across all resting periods (*p* < 0.0001). (2) DM + ZnSO_4_ vs. DM: Zinc treatment resulted in a statistically significant functional improvement in all parameters across all resting periods (*p* < 0.05, *p* < 0.01, and *p* < 0.0001). (3) CON + ZnSO_4_ vs. CON: Zinc supplementation in healthy rats caused a highly significant reduction in mechanical contraction parameters (CF, +dF/dtmax, −dF/dtmax) compared with the CON group at all waiting times (*p* < 0.0001); however, for the AUC parameter, no statistically significant reduction was observed at 10 s, 40 s, and 60 s resting times (*p* > 0.05). (4) DM + ZnSO4 vs. CON: There was no statistically significant difference in mechanical contraction parameters (*p* > 0.05), indicating functional restoration to near-control levels, except for +dF/dt_max_ and −dF/dt_max_ at 10s and 20s, which remained partially lower (*p* < 0.05). (5) Comparisons between CON + ZnSO_4_ and DM groups showed no significant differences (*p* > 0.05) across most parameters.

A post-rest potentiation protocol involving rest periods of 10, 20, 30, 40, 50, and 60 s was applied in an isolated organ bath to evaluate the calcium dynamics of the sarcoplasmic reticulum (SR) in diaphragm muscle strips. The parameters CF, +dF/dtmax, −dF/dtmax, and AUC were analyzed from the isometric contraction recordings (Figure 5).

Across all rest periods applied (ranging from 10 to 60 s), a statistically significant reduction in CF, +dF/dtmax, -dF/dtmax, and AUC values was observed in the DM group compared with the CON group (*p* < 0.0001). In contrast, in the DM + ZnSO_4_ group, where ZnSO_4_ treatment was administered to the diabetic model, all impaired mechanical contraction parameters were found to have improved and increased to a statistically significant extent compared with the DM group (*p* < 0.05, *p* < 0.01, and *p* < 0.0001). These data indicate that ZnSO_4_ administration has a therapeutic effect on the mechanical dysfunction of the diaphragm muscle caused by DM.

On the other hand, when the contraction parameters (CF, ±dF/dtmax, and AUC) of the healthy CON + ZnSO_4_ group, which received zinc supplementation, were evaluated, a statistically significant decrease was observed compared with the CON group at all waiting times (*p* < 0.0001).

Notably, a highly significant and unexpected finding of this study was observed in the healthy zinc-treated group (CON + ZnSO_4_). Daily zinc supplementation in healthy control animals led to a profound and systemic depression of almost all diaphragmatic mechanical contraction parameters compared with the untreated CON group. Specifically, across all post-rest resting periods (10, 20, 30, 40, 50, and 60 s), the CON + ZnSO4 group exhibited highly significant reductions in contraction force (CF), maximum contraction velocity (+dF/dtmax), and maximum relaxation velocity (−dF/dtmax) (*p* < 0.0001). Contraction power (AUC) was also significantly depressed at 20, 30, and 50 s of resting times (*p* < 0.0001). This marked reduction indicates that excess exogenous zinc under non-pathological conditions may exert a depressive or inhibitory effect on diaphragmatic muscle mechanics, possibly by interfering with physiological calcium dynamics, ryanodine receptor kinetics, or cross-bridge cycling.

## 4. Discussion

In addition to the chronic systemic effects it causes, DM is a metabolic disease that leads to serious structural and functional impairments in skeletal muscles and, particularly in mammals, in the diaphragm—the primary muscle involved in respiration [19]. Cellular damage triggered by hyperglycemia causes both histological abnormalities and mechanical contraction deficits in the diaphragm muscle [20,21,22]. In this study, we investigated the histopathological, immunohistochemical, and functional dysfunctions occurring in the diaphragm muscle of an aged diabetic rat model, as well as the potential effects of exogenous ZnSO_4_ treatment on these parameters. The findings indicate that while zinc treatment cannot reverse histological tissue damage such as apoptosis and fibrosis in the diabetic diaphragm, it does provide a significant improvement in mechanical contraction function by preserving the expression of *SERCA2a*, a calcium regulator of the sarcoplasmic reticulum (SR).

Chronic hyperglycemia and cellular oxidative stress, which underlie diabetic skeletal muscle damage, disrupt tissue architecture and exacerbate apoptosis and pro-fibrotic processes [8]. Upon examination of the histopathological findings of our study, diaphragm sections from the DM group revealed distinct muscle fiber disorganization, vacuolization, inflammatory leukocyte infiltration, and fibrotic foci indicating an increase in connective tissue. These histological findings, which indicate a disruption of structural integrity, are fully consistent with the intense immunohistochemical staining of *Caspase-3*—a key marker of cellular apoptosis—and the marked increase in the gene expression of *Caspase-3* and *TGF-beta1*, as determined by qPCR analysis. Various studies in the literature have reported that zinc supplementation successfully suppresses *TGF-beta1* production and exhibits anti-apoptotic effects by activating antioxidant pathways (particularly Nrf2) in models of diabetic cardiomyopathy, nephropathy, and liver damage [8,23]. However, in our study, signs of inflammation and fibrosis persisted in the diaphragm sections of the DM + ZnSO_4_ group, and no statistically significant reduction was observed in *Caspase-3* and *TGF-beta1* mRNA levels.

This tissue-specific discrepancy and the failure of ZnSO_4_ to suppress these markers in the diaphragm, despite positive outcomes in other tissues, can be attributed to several unique physiological factors. First, unlike relatively quiescent metabolic organs like the liver or resting skeletal muscles, the diaphragm is a continuously active respiratory pump under unrelenting mechanical workload. Chronic diabetic glucotoxicity combined with this continuous contractile strain creates an extremely hostile, high-turnover cellular environment that perpetually stimulates inflammatory and apoptotic cascades, thereby overwhelming the transcriptional anti-apoptotic capacity of zinc [24]. Second, our study utilized 18-month-old female rats modeling postmenopausal ovarian senescence. Advanced age and estrogen depletion independently accelerate mitochondrial decay, cellular senescence, and progressive proteostotic decline in skeletal muscle [25]. Once the pro-fibrotic (TGF-beta1) and apoptotic (*Caspase-3*) pathways are chronically active and structurally established in an aged, sarcopenic diaphragm, a short-term 6-week zinc intervention is insufficient to transcriptionally reverse or silence these established structural remodeling processes, even though it successfully rescues functional calcium dynamics via *SERCA2a*. Third, intracellular zinc homeostasis in skeletal muscle is highly dependent on specific transporter kinetics, such as ZIP7 (Slc39a7) and ZnT transporters [26]. Chronic diabetic injury and high-fat diet pre-treatment have been documented to downregulate crucial skeletal muscle zinc importers like ZIP7, which restricts the intracellular accumulation of free zinc ions [26]. Consequently, the therapeutic intracellular zinc threshold required to directly bind and inhibit apoptotic executioners like *Caspase-3* or to suppress the TGF-beta1 promoter may not have been achieved in the diaphragmatic myofibers, leaving these destructive pathways uninhibited, in contrast to metabolic tissues with different zinc transport and accumulation kinetics.

Despite the persistence of structural damage at the histopathological level, findings related to the diaphragm’s primary function—mechanical contraction—demonstrated that zinc successfully supported cellular mechanics. In our isolated organ bath recordings, a significant decrease was observed in the DM group in terms of the baseline contraction force (CF), the area under the curve (AUC), and the ±dF/dtmax values reflecting contraction and relaxation kinetics. This mechanical weakness in skeletal muscles is consistent with previous studies highlighting that diabetes causes muscle dysfunction [27]. The decrease in relaxation rate (−dF/dtmax) is directly associated with dysfunction of SR calcium reuptake mechanisms and the SERCA pump [28]. Our PCR analyses demonstrated that *SERCA2a* gene expression was significantly suppressed in the diabetic model; meanwhile, ZnSO_4_ administration prevented this suppression in the DM + ZnSO_4_ group, thereby preserving *SERCA2a* levels. Zinc’s protective role in preserving these regulatory proteins that maintain calcium homeostasis is closely linked to its support of intracellular antioxidant defense. Indeed, it has been reported that zinc deficiency exacerbates diabetic complications by increasing oxidative stress [29], and that zinc is an essential element for Nrf2 transcriptional function in tissues such as the kidneys and liver under T2DM conditions [30,31]. Therefore, the basis for the functional rescue observed in our study—despite impaired cellular architecture—lies in zinc’s role in preserving intracellular calcium dynamics (*SERCA2a*) and sustaining muscle contractile mechanics.

To functionally validate whether the transcriptional preservation of *SERCA2a* translates into physiological improvements in calcium handling, we utilized a post-rest potentiation (PRP) protocol (with rest intervals from 10 to 60 s) in our isolated organ bath experiments. In respiratory muscle biophysics, the recovery of contraction force and the velocity of relaxation (−dF/dt_max_) following rest intervals are directly dependent on the sarcoplasmic reticulum (SR) calcium load. During these stimulation-free rest intervals, the cytosolic free Ca^2+^ is sequestered back into the SR lumen—a process driven almost entirely by the active transport of the *SERCA2a* pump in diaphragmatic tissue. Therefore, the significant, rest-interval-dependent recovery of contractile parameters observed in the DM + ZnSO_4_ group functionally confirms that the preserved transcriptional levels of *SERCA2a* are directly associated with a restored and fully operational SR calcium recycling machinery.

Another key finding is the significant contractility decrease in healthy zinc-supplemented controls (CON + ZnSO_4_). Trace element homeostasis in healthy tissues requires a delicate balance [25]. Excessive skeletal muscle zinc accumulation can impair excitation-contraction (E-C) coupling by directly inhibiting sarcoplasmic reticulum ryanodine receptor (RyR1) channels [32]. This limits cytosolic Ca^2+^ release upon depolarization, directly reducing force generation (CF and AUC). Additionally, while zinc classically acts as an antioxidant, excessive accumulation in healthy tissues can paradoxically trigger a pro-oxidant state, as previously noted in this cohort’s myocardium [13]. Excess mitochondrial zinc disrupts the electron transport chain, increasing reactive oxygen species (ROS) production. This localized oxidative stress damages contractile proteins, impairs myosin ATPase, and oxidizes *SERCA2a* sulfhydryl groups, leading to the observed velocity and contractility deficits.

While this study demonstrates a clear correlation between functional diaphragmatic contractility and the transcriptional preservation of *SERCA2a* following zinc supplementation, some limitations must be acknowledged. First, our evaluation of calcium homeostasis was restricted to quantitative mRNA gene expression of *SERCA2a* and functional post-rest potentiation (PRP) parameters. Direct measurements of *SERCA2a* protein levels, SERCA pump activity, or real-time intracellular Ca^2+^ dynamics were not performed. Second, our histological assessment of fibrosis and apoptosis was based on semi-quantitative scoring of H&E and active *Caspase-3* immunohistochemistry. Although these evaluations were performed in a blinded, structured manner and strongly corroborated by quantitative qPCR data (*TGF-beta1* and *Caspase-3* mRNA), the lack of histochemical collagen-specific stains (such as Masson’s Trichrome or Sirius Red) and in situ end-labeling assays (such as TUNEL) represents a limitation of the current study. Third, although the combination of HFD and low-dose STZ is a widely accepted model for T2DM, direct measurements of serum insulin, C-peptide, or insulin resistance indices (such as HOMA-IR) were not performed in this specific cohort, meaning the exact degree of peripheral insulin resistance remains unquantified. Fourth, we did not measure local diaphragmatic tissue zinc levels to biochemically confirm the exact intracellular accumulation and efficacy of our supplementation protocol. Fifth, although we discuss zinc’s protective role primarily through its well-established antioxidant capacity, direct tissue oxidative stress markers (such as malondialdehyde, superoxide dismutase, or glutathione) were not assessed in the diaphragm to biochemically validate this specific protective mechanism. Finally, our study utilized exclusively female aged rats to model postmenopausal metabolic changes, which limits the immediate generalizability of these findings to male cohorts. Future work incorporating these specific histological, calcium imaging, metabolic characterization, tissue element analysis, and oxidative stress assays across both sexes will provide deeper qualitative insight into the structural and metabolic remodeling of the diabetic diaphragm.

## 5. Conclusions

In conclusion, this study demonstrates that, although ZnSO_4_ treatment in an aged diabetic rat model cannot reverse the pro-apoptotic, inflammatory, and fibrotic processes occurring in the diaphragm muscle at the structural (histological) level, it demonstrates that ZnSO_4_ treatment significantly improves the muscle’s mechanical contraction dysfunction at the functional level by preserving SR calcium homeostasis (*SERCA2a*). These cellular effects of zinc—which acts as a protective agent that improves functional capacity under pathological conditions and as an element that modulates physiological responses under healthy conditions—emphasize the importance of preserving calcium regulators in the treatment of respiratory muscle weakness secondary to diabetes. Consequently, future research should focus on validating these findings at the protein level using Western blot or ELISA assays for *SERCA2a*, *TGF-beta1*, and *Caspase-3*. Additionally, conducting quantitative tissue element analysis to measure local zinc levels, assessing direct oxidative stress biomarkers (such as MDA, SOD, and catalase), and performing real-time calcium imaging on isolated diaphragmatic fibers will provide deeper biophysical insights. Finally, comparative studies utilizing both male and female cohorts, along with estrogen rescue designs, are warranted to fully clarify the sex-dependent therapeutic index of zinc supplementation in diabetic skeletal muscle disorders.

## Figures and Tables

**Table 1:** Primers used for qPCR.

Gene	Forward Primer Sequence (5′→3′)	Reverse Primer Sequence (5′→3′)
*SERCA2a*	CAGAGAGACGCCTGCTTAAAT	GTTCACACCATCACCAGTCATA
*Caspase-3*	CCACGGAATTTGAGTCCTTCT	CCACTCCCAGTCATTCCTTTAG
*TGF-β1*	CTGAACCAAGGAGACGGAATAC	CGGGTTGTGTTGGTTGTAGA
*GAPDH*	AACTCCCTCAAGATTGTCAGCAA	GGCATGGACTGTGGTCATGA

**Table 2:** Body weight and fasting blood glucose (FBG) parameters of the experimental groups.

Parameters	CON (n = 8)	CON + ZnSO_4_ (n = 8)	DM (n = 8)	DM + ZnSO_4_ (n = 8)
Initial Body Weight (g)	245.5 ± 8.2	242.3 ± 7.9	248.1 ± 9.4	246.7 ± 8.8
Final Body Weight (g)	275.8 ± 10.4	270.2 ± 9.1	210.4 ± 11.2 *	235.6 ± 9.8 *^#^
Initial FBG (mg/dL)	105.3 ± 5.2	103.8 ± 4.9	106.1 ± 6.0	104.7 ± 5.5
FBG at Week 4 (post-STZ) (mg/dL)	106.4 ± 4.8	104.2 ± 5.1	350.2 ± 18.4 *	345.8 ± 16.9 *
Final FBG at Week 14 (mg/dL)	108.2 ± 6.1	105.1 ± 4.5	395.4 ± 22.8 *	262.1 ± 19.3 *^#^

Data are presented as mean ± SD. * *p* < 0.001 vs. CON group; # *p* < 0.01 vs. DM group.

## Data Availability

The original contributions presented in this study are included in the article. Further inquiries can be directed to the corresponding author.
